# Radiation‐Based 3D Dual‐Mode Thermal Management Devices: Advances in Active/Passive Switching for Energy‐Saving Applications

**DOI:** 10.1002/smll.202514437

**Published:** 2026-04-24

**Authors:** Su Eon Lee, Ho Jun Jin, Jun Hyun Park, Ha Uk Chung, Junyong Seo, Bong Hoon Kim

**Affiliations:** ^1^ Department of Robotics and Mechatronics Engineering DGIST Daegu Republic of Korea; ^2^ Center for Nano Material Development National Nanofab Center (NNFC) Daejeon Republic of Korea; ^3^ School of Biomedical Engineering Korea University Seoul Republic of Korea; ^4^ Department of Mechanical and Automotive Engineering Kongju National University Cheonan Republic of Korea; ^5^ Global Institute of Manufacturing Technology (GITECH) Kongju National University Cheonan Republic of Korea; ^6^ Department of Future Convergence Engineering Kongju National University Cheonan Republic of Korea; ^7^ Center for Physical AI DGIST Daegu Republic of Korea; ^8^ TransHuman Robotics Research Center (Glocal Lab) DGIST Daegu Republic of Korea; ^9^ Global Bio‐integrated Materials Center (Engineering Research Center) KAIST Daejeon Republic of Korea

**Keywords:** dual‐mode thermal management devices, electronics, optics, radiative cooling, solar heating

## Abstract

Radiation‐based energy‐saving strategies, including solar heating (SH) and radiative cooling (RC), have emerged as sustainable solutions that operate without an external energy input. SH relies on broadband solar absorbers to capture solar radiation, whereas RC employs selective emitters with high solar reflectance and strong thermal emissivity in the atmospheric transparent window regime. Despite their potential, single‐mode SH or RC devices lack adaptability under dynamic environmental conditions. To overcome these limitations, dual‐mode devices that integrate both SH and RC have been proposed. However, 2D film‐type dual‐mode devices have limited tunability and environmental adaptability. This perspective paper highlights the development of 3D dual‐mode thermal management devices that exploit structural deformations—axial loading, flipping, and bending—for reversible switching between SH and RC. Active devices enable dynamically adaptive control, whereas passive devices achieve zero‐energy operation. Overall, 3D dual‐mode devices represent a promising platform for sustainable energy savings. This paper reviews the recent advances in this field, classifies the devices according to the deformation mode, and discusses existing challenges and future opportunities. Thus, it serves as a comprehensive guide for researchers and industry professionals, inspiring future innovations and contributing to sustainable thermal regulation strategies.

## Graphical abstract

Heating and cooling energy demand account for nearly half of the global energy consumption. SH and RC offer sustainable, zero‐energy strategies, but single‐mode devices lack adaptability. 3D dual‐mode devices, exploiting axial, flipping, and bending deformations, provide reversible switching with dynamic or passive control, representing a promising platform for efficient and sustainable energy savings.

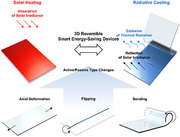

## 1 Introduction

Heating and cooling loads are the largest contributors to the energy demand of a building, accounting for nearly half of the worldwide energy consumption [1, 2]. Driven by climate change and population growth, the demand for heating, ventilation, and air conditioning (HVAC) systems in buildings and vehicles is expected to rise annually by approximately 6.2% [3]. The corresponding growth in energy demand is anticipated to intensify critical environmental issues, including global warming and air pollution. To address these challenges, radiation‐based energy‐saving strategies, such as solar heating (SH) and radiative cooling (RC), have attracted increasing attention as future thermal management solutions that operate without an external energy input [4, 5, 6, 7, 8, 9, 10]. Research on SH has focused on broadband solar absorbers (BAs) capable of absorbing the broad spectral radiation emitted from the sun (≈ 5800 K) [11, 12, 13, 14, 15, 16, 17]. Notable examples include carbon nanotubes (CNTs) [18, 19, 20], MXene [21, 22, 23, 24], and carbon‐based blackbody structures [25, 26, 27, 28, 29]. By contrast, RC functions by dissipating heat into outer space through the atmospheric transparent window (8–13 µm) [30, 31, 32, 33, 34]. This is achieved using selective emitters (SEs) that combine a high solar reflectance (0.3–2.5 µm) with strong thermal emissivity (8–13 µm) [35, 36, 37, 38, 39]. Examples include polyethylene oxide nanofibers [37], mesoporous cellulose nanofibers (CNFs) [33], and stereoscopic oligosiloxane–based polyurea structures [40]. By contrast, RC functions for a daytime sub‐ambient cooling by dissipating heat into outer space through the atmospheric transparent window (8–13 µm) [30, 31, 32, 33, 34]. This is achieved using selective emitters (SEs) that combine a high solar reflectance (0.3–2.5 µm) with strong thermal emissivity (8–13 µm) [35, 36, 37, 38, 39]. Examples include polyethylene oxide nanofibers [37], mesoporous cellulose nanofibers (CNFs) [33], and stereoscopic oligosiloxane–based polyurea structures [40]. Note that, enhancing broadband emission in the overall infrared regime (i.e., 5–50 µm) is also regarded as the strategy to improve radiative cooling effect. However, we have focused on the energy‐saving application of the RC devices. So that, enhancing emission in only atmospheric transparent window regime is more preferred due to its daytime, sub‐ambient cooling performance. Despite their environmentally friendly and sustainable nature, which enables a high heating or cooling performance, these single‐mode approaches have limited practical applicability. Devices that provide only heating or only cooling lack adaptability to diverse environmental conditions.

To overcome these constraints, dual‐mode devices capable of integrating both heating and cooling within a single platform have recently been proposed [41, 42, 43, 44, 45]. Dual‐mode thermal management refers to the integration of SH and RC and their reversible switching to accommodate varying ambient conditions. Unlike static single‐mode systems, the 3D architectures reviewed in this paper utilize reversible mechanical deformations—such as axial loading, bending, or flipping—as mechanisms for mode transitions. By dynamically modulating the effective exposure area and the optical view factor of the SH‐ and RC‐dominant functional layers, these stereoscopic configurations overcome the geometric constraints of planar films and enable environment‐adaptive thermal regulation. These approaches have been reported to achieve up to 12.4% higher energy savings when compared with that of single‐mode systems [46, 47, 48], particularly under real‐world conditions characterized by large diurnal temperature fluctuations or distinct seasonal variations. For example, in the Republic of Korea, energy savings of 4.9%–6.8% during summer (June–August) and 4.9%–5.6% during winter (December–February) have been demonstrated [46]. Beyond HVAC [49], dual‐mode devices show potential for enhancing the thermal efficiency in diverse applications, including optoelectronic devices [50, 51, 52], sensors [53, 54, 55], energy systems [56, 57, 58], biomedical devices [59, 60, 61], actuators [62, 63], and microfluidic platforms [64, 65, 66]. In addition, polydimethylsiloxane (PDMS)‐based systems have been investigated to design dual‐mode thermal management devices adaptable to variable environmental conditions [67]. For instance, PDMS mixed with graphene nanopowder served as a BA, achieving a high solar absorptance of 93.6%, whereas PDMS micro–nano fiber used as an SE exhibited a solar reflectance of 93%. By combining these complementary features, a device capable of modulating the heating and cooling performance through mechanical compression was demonstrated [68]. In another study, a carbon black layer, functioning as a blackbody BA with a low solar reflectance of 2.8%, was integrated with a cellulose acetate aerogel SE, which exhibited both high solar reflectance (95.7%) and strong long‐wave infrared (LWIR) emissivity (93%). This bilayer structure was used to realize a device that performed both SH and RC [69]. These smart energy‐saving devices provide a promising strategy for reducing global energy consumption. However, significant challenges remain in realizing their practical implementation across diverse fields. For example, dual‐mode devices fabricated in the form of 2D films exhibit limited adaptability to variations in solar irradiance and restricted tunability of radiative properties. For example, dual‐mode devices fabricated in the form of 2D films often exhibit limited adaptability and restricted tunability of radiative properties because their geometry is intrinsically static, making it difficult to respond to dynamic outdoor conditions such as varying sunlight incidence and azimuth angles. As a result, 2D implementations frequently rely on external mechanical or electrical components to trigger mode transitions, introducing a non‐negligible switching overhead in terms of auxiliary energy input and system complexity. Furthermore, within a fixed footprint, 2D configurations suffer from an inherent area‐efficiency trade‐off, meaning the projected aperture must be shared between SH‐ and RC‐dominant regions, significantly limiting the achievable modulation depth of the effective radiative response and confining operation to suboptimal—or effectively single‐mode—regimes.

To overcome these limitations, the development of 3D dual‐mode thermal management devices—capable of flexibly responding to dynamic environmental conditions and modulating the thermal performance through structural mechanical deformations—has become essential. Specifically, 3D architectures provide additional design freedom via out‐of‐plane deformations, enabling reversible and adaptive switching between SH‐ and RC‐dominant states through temperature‐responsive actuation (e.g., shape‐memory effects). These stereoscopic transformations allow dynamic modulation of the effective exposure area and the radiative view factor, making efficient solar collection or shading easier to achieve than with static planar structures. Furthermore, as demonstrated in several recent studies, by leveraging partially decoupled 3D motions from the substrate, such devices can deliver more precise and rapid thermal responses to fluctuating outdoor conditions, including variations in solar intensity and ambient temperature. Consequently, these structural advantages can improve robustness and help maintain stable thermal management performance under diurnal and seasonal weather variations. To clarify the scope of this perspective paper, we define 2D devices as thin‐film‐based structures that maintain a constant planar configuration without significant out‐of‐plane deformation during operation. Specifically, even when subjected to temperature‐induced material property modulations or microscopic thermal expansion, these devices are classified as 2D if their overall geometry does not transition into a stereoscopic architecture, thereby keeping the effective exposure area and radiative view factor largely constant. In contrast, 3D devices refer to systems that undergo macroscopic shape transformations through spontaneous or externally induced out‐of‐plane motions, such as bending, flipping, or axial deformation. Consequently, these devices enable dynamic, temperature‐dependent modulations of their effective radiative behavior by actively reconfiguring their geometric parameters, including the exposure area and the radiative view factor.

In this perspective paper, we explore 3D dual‐mode thermal management devices, classifying them into active and passive systems, and further categorizing each device according to the mechanical deformation mode for a detailed analysis. This approach is motivated by three key considerations: (i) owing to their switching mechanisms, active devices can serve as practical, dynamically adaptive, and radiative thermal management solutions under variable environmental conditions; (ii) passive devices enable zero‐energy‐consumption performance by relying solely on radiative control without requiring an external energy input; (iii) classifying the devices according to the deformation mode—axial loading, flipping, or bending—highlights their potential as platform technologies capable of integrating the high‐performance SH/RC functionalities achieved by single‐mode systems. In this paper, we review the latest applications of practical and efficient energy‐saving solutions, systematically organize and classify the devices, and finally discuss the existing challenges and future directions. Overall, this perspective paper aims to serve as a comprehensive and up‐to‐date reference for researchers and industry professionals, thereby fostering advances and innovations in radiation‐based 3D dual‐mode thermal management devices, accelerating their real‐world implementation, and ultimately contributing to sustainable thermal regulation and mitigation of global environmental issues.

## 2 Principles of SH and RC for Radiation‐Based 3D Dual‐Mode Thermal Management Devices

To realize radiation‐based SH, it is essential to design a broadband solar thermal absorber capable of effectively harvesting the incident solar radiation. The design goal can be achieved by maximizing the average solar absorptance (*Ā_solar_)*, which is defined as follows:

(1) $${\bar{A}}_{solar}=\frac{\int_{0.3\;\mu m}^{2.5\;\mu m}I_{solar}\left(\lambda \right)\cdot \alpha \left(\lambda \right)\;d\lambda }{\int_{0.3\;\mu m}^{2.5\;\mu m}I_{solar}\left(\lambda \right)\;d\lambda }\;$$

Here, 𝜆 denotes the wavelength, *I*_s__o__l__a__r_(𝜆) denotes the solar irradiance, and α(𝜆) denotes the spectral absorptance of the sample. Such broadband solar thermal absorbers are designed to mimic blackbody‐like absorption characteristics across the solar spectrum, utilizing materials with high intrinsic absorption, such as carbon‐ or MXene‐based systems. For example, under the AM 1.5G solar spectrum, carbonized melamine foam achieves 96.8% light absorption over the full wavelength range [70, 71], whereas MXene–SiO_2_–Fe metasurfaces reach an absorption rate exceeding 90% [72]. Consequently, extensive research efforts have been directed toward achieving high radiative energy absorption using these approaches.

In contrast, RC can be achieved by maximizing the RC power (*P_cool_*), which is defined as follows: [34, 73, 74]

(2) $$P_{cool}=P_{rad}\;-P_{sol}-P_{atm}$$

(3) $$P_{rad}=\int_{0}^{\infty }\varepsilon \left(\lambda \right)I_{b}\left(\lambda ,T_{amb}\right)d\lambda \;$$

(4) $$P_{sol}=\int_{0}^{\infty }\alpha \left(\lambda \right)I_{solar}\left(\lambda \right)d\lambda \;$$

(5) $$P_{atm}=2\int_{0}^{\infty }\int_{0}^{\frac{\pi }{2}}\alpha \left(\lambda \right)I_{b}\left(\lambda ,T_{amb}\right)\left(1-\tau_{atm}{\left(\lambda \right)}^{\frac{1}{\cos\theta }}\right)\cos\theta \sin\theta d\theta d\lambda$$

Here, *P_rad_* represents the thermal radiation spontaneously emitted from the sample with temperature *T_amb_*, *P_sol_* is the absorbed solar irradiance, and *P_atm_* denotes the absorbed atmospheric radiation. In Equation (3), *ε(λ)* is the spectral emissivity of the sample, *I_b_(λ, T)* is the spectral intensity of blackbody radiation at a temperature *T* (i.e., Planck's distribution), and *T_amb_* is the ambient temperature [75]. The notations used in Equation (4) are the same as those in Equation (1); *α(λ)* in this case is physically equivalent to the spectral emissivity. In Equation (5)*, τ_atm_(λ)* denotes the wavelength‐dependent atmospheric transmission spectrum, and *θ* is the polar angle at which the atmosphere is viewed from the sample surface. To calculate the standardized radiative cooling power of a sample, it is a common practice to evaluate *P_cool_* in Equation (2) by regarding the sample's surface temperature in Equation (3) as *T_amb_*. To maximize RC performance (i.e., to achieve a high *P_cool_*), it is essential to design an SE that can effectively reflect the incoming solar radiation in the solar spectrum (0.3–2.5 µm) to lower *P_sol_*, while simultaneously enabling strong thermal emission in the midinfrared (MIR) range (8–13 µm) to maximize *P_rad_* and minimize *P_atm_*. For example, a fibrous membrane composed of polyvinylidene fluoride/tetraethyl orthosilicate fibers containing numerous internal nanopores and randomly distributed SiO_2_ microspheres on the surface exhibits an average mid‐infrared (MIR, 8–13 µm) emissivity exceeding 0.96 and reflects approximately 97% of the incident solar radiation. Under a solar irradiance of 1000 W/m^2^, this structure achieved an average RC power of 61 W/m^2^ and a maximum temperature reduction of 6°C [76]. As another example, an electrospun polyethylene oxide film exhibited a selective emissivity of 78% in the 8–13 µm wavelength range and achieved a high reflectance of 96.3% in the 0.3–2.5 µm range through a diameter‐controlled nanofiber structure. Consequently, an additional cooling of approximately 3°C in comparison with that of non‐SEs was observed at night, whereas under daytime solar illumination, sub‐ambient cooling of approximately 5°C below the ambient temperature was realized [37]. Even though the averaged emissivity at 8–13 µm was relatively low compared to the broadband (i.e., non‐selective) emitter, lower cooling temperature of the nanofiber structure was achieved by blocking the incident ambient emission through its strong selectivity [37].

**FIGURE 1:**
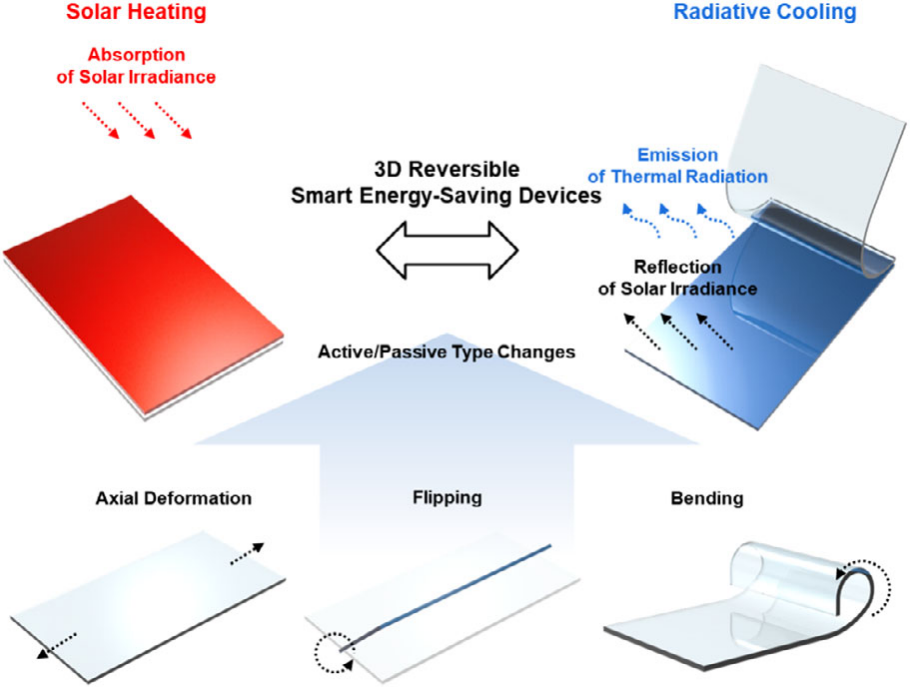
Schematic of radiation‐based 3D dual‐mode thermal management devices, showing the principles of SH and RC, as well as the reversible switching mechanisms (e.g., axial deformation, flipping, and bending) that enable dynamic control of the thermal states under environmental variations.

By integrating these high‐performance SH and RC components, radiation‐based 3D dual‐mode thermal management devices enable reversible switching between the two modes, thereby facilitating broader practical applications. Such systems can be employed in a wide range of fields, including HVAC systems, optics, sensors, energy devices, biomedical devices, actuators, and microfluidic platforms. 3D dual‐mode thermal management devices exhibit high adaptability to dynamic environments—such as variations in solar irradiance, shadow formation, and diurnal temperature fluctuations—and are thus promising as energy‐saving solutions. As illustrated in Figure 1, reversible switching mechanisms can be broadly classified into active and passive actuations, which are based on deformation principles such as axial deformation, flipping, and bending. The detailed mechanisms of these approaches are discussed in the following section.

## 3 Active‐Type 3D Dual‐Mode Thermal Management Devices

### 3.1 Axial Deformation

**FIGURE 2:**
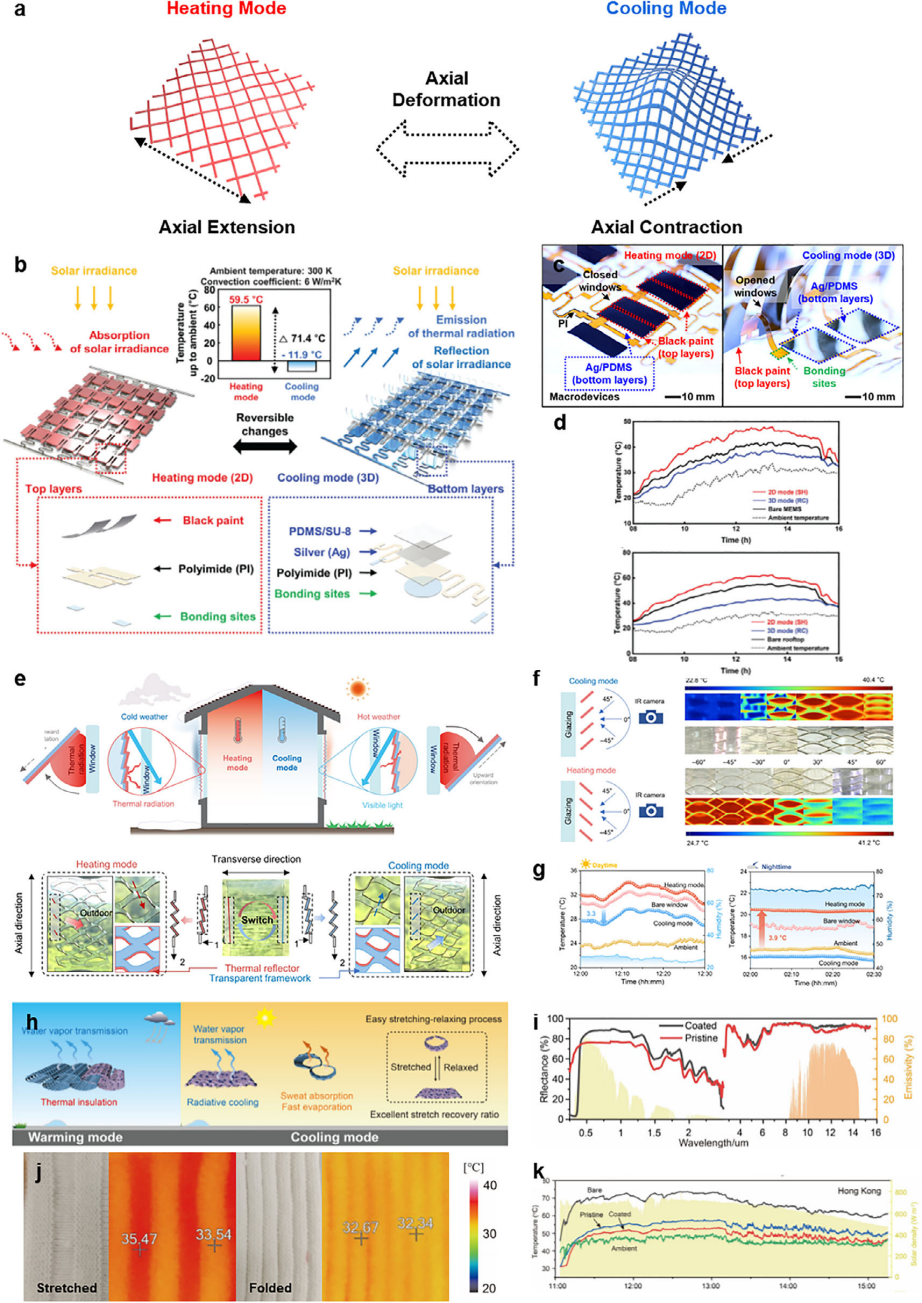
a) Schematic illustration of radiation‐based 3D dual‐mode thermal management devices actuated by active axial deformation. b) Schematic of mechanical buckling‐based 3D dual‐mode thermal management devices. c) Photographs of the devices in the heating and cooling modes. d) Experimental thermal performances of the devices measured on MEMS platforms and rooftops. Reproduced with permission [67]. Copyright 2024, WILEY. e) Schematics and photographs of kirigami‐structured 3D dual‐mode thermal management devices. f) Optical and IR images of the devices in heating and cooling modes. g) Daytime and nighttime thermal performances of kirigami‐based devices. Reproduced under terms of the CC‐BY license [78]. Copyright 2023, H. Yin et al., published by AAAS. h) Schematic of the 3D self‐folding fabric and its SH and RC mechanisms. i) Optical properties of coated versus pristine fabrics. j) Optical and IR images of stretched and folded 3D self‐folding fabrics. k) Experimental thermal performances of the 3D self‐folding fabrics under outdoor conditions. Reproduced under terms of the CC‐BY license [79]. Copyright 2025, X. Zhang et al., published by Springer Nature.

Active 3D dual‐mode thermal management devices have attracted significant attention as efficient energy‐saving solutions, particularly in regions with large seasonal variations or pronounced diurnal temperature fluctuations, as they preserve the advantages of conventional single‐mode SH/RC while offering enhanced adaptability to dynamic environments. For example, through instantaneous structural reconfiguration, these devices can suppress excessive RC that may occur during winter nights, thereby enabling stable temperature regulation under diverse environmental conditions [77]. Among the active deformation strategies, the simplest mode‐switching approach is axial deformation (Figure 2a). As shown in Figure 2b, reversible transitions between the 2D (heating mode) and 3D (cooling mode) states can be achieved through uniaxial stretching of a silicone elastomer [67]. The constituents of the top layer of the device are black paint, polyimide (PI), and bonding sites, whereas those of the bottom layer are PDMS (for macrodevices) or SU‐8 (for microdevices) along with Ag, PI, and bonding sites. As illustrated in Figure 2c, the black paint on the top layer functions as a BA. Its absorptance approaches unity across the entire solar spectrum, thereby enabling effective SH. In contrast, the Ag/PDMS or SU‐8/PDMS bottom layer serves as an SE, promoting RC through low solar absorptance and high emissivity within the atmospheric transparency window (8–13 µm). This device is fabricated through a mechanical buckling process, enabling reversible transitions between the 2D and 3D configurations under uniaxial strains of up to 100%. By incorporating microfabrication techniques, devices with a wide range of length scales can be realized, from the microscale to several centimeters. In particular, the tilt angle of the top layer can be tuned according to the degree of uniaxial deformation, thereby allowing continuous control over the SH and RC performance. Experimentally, a temperature regulation of +4.8/−4.2°C (MEMS device) and +5.3/−11.0°C (rooftop installation) relative to the ambient temperature was demonstrated (Figure 2d), as measured at 14:00 on May 24, 2024, in Daejeon, Republic of Korea (36.38°N, 127.36°E). Furthermore, simulations confirmed that reversible heating and cooling performance can be achieved on various substrates, including skin, glass, steel, aluminum, copper, and PI.

Figure 2e illustrates an active‐type 3D dual‐mode thermal management device based on a kirigami structure [78]. The kirigami envelope was fabricated using indium tin oxide (ITO) and polyethylene terephthalate (PET), both exhibiting high transparency in the visible spectrum, together with PDMS as the framework material to ensure mechanical stability. The multilayer thin film (ITO/PET/PDMS) was laser‐cut into a kirigami pattern and subsequently integrated with glazing to complete the device. The glazing, serving as an emitter, exhibited a high LWIR emissivity (*ε_LWIR_*) of 0.91, whereas the ITO reflector showed a very low *ε_LWIR_* of 0.07. Axial deformation induced mechanical instabilities in the kirigami envelope, leading to out‐of‐plane deformations in which the tilt angle of the bridges varied under the applied tensile strain. This mechanism enabled active and reversible switching between the heating and cooling modes. In the heating mode, the bridges tilt downward, orienting the ITO reflective layer toward the glazing to suppress radiative heat loss. Conversely, in the cooling mode, the bridges tilt upward, positioning the ITO layer upward to maximize heat dispersion through radiative emission. After being switched to either the heating or cooling mode through tensile deformation, the kirigami envelope was attached to a glazing surface preheated to 40°C (Figure 2f), and the surface temperature was subsequently monitored simultaneously from different viewing angles. In the heating mode, as the observation angle increased counterclockwise, the apertures became progressively covered, resulting in IR images with blue‐shifted coloration, indicating that thermal radiation from the window was effectively blocked in that direction. In contrast, in the cooling mode, additional apertures were exposed as the observation angle increased, and the images shifted toward red, clearly demonstrating unimpeded radiative heat emission. Furthermore, under daytime solar irradiance exceeding 720 W/m^2^, the envelope in the cooling mode maintained a surface temperature approximately 3.3°C lower than that of an untreated window, demonstrating a distinct cooling effect, whereas at night, it remained below the ambient temperature, confirming RC performance. Conversely, in the heating mode, the envelope maintained a higher temperature during the day than that observed with the untreated window and effectively suppressed heat loss at night, sustaining a temperature that was approximately 3.9°C above the ambient temperature (Figure 2g).

Figure 2h presents an active‐type 3D dual‐mode thermal management device with a self‐folding structure [79]. This fibrous device is fabricated by coating cotton yarn with TiO_2_ nanoparticles and PDMS. The TiO_2_ nanoparticles enhance the solar reflectance by inducing light scattering, whereas PDMS provides mechanical flexibility together with excellent IR emissivity. The TiO_2_/PDMS/cotton‐based device exhibited a high solar reflectance (*R_solar_*) of 89.5% (compared to 78% for the pristine fabric) and an *ε_LWIR_* of 93.5% within the atmospheric transparency window (8–13 µm) (Figure 2i). This fabric enables reversible switching between the heating and cooling modes through axial deformation. In the undeformed state, the intrinsic 3D self‐folded structure of the textile is maintained, creating trapped air layers that suppress heat conduction and convection, whereas the bulk thickness provides thermal insulation, thereby realizing the heating mode. In contrast, when the textile is stretched into a planar configuration, its high solar reflectance and LWIR emissivity promote radiative heat dissipation, resulting in the manifestation of the cooling mode via RC. Figure 2j presents the optical and thermal images of the pristine self‐folded fabric (right, 3D mode) and stretched fabric (left, 2D mode) placed on a hot plate. A temperature difference exceeding 1°C was observed between the two structures, demonstrating the clear thermal insulation capability of the 3D fabric. In addition, Figure 2k experimentally shows that, under solar irradiation, the stretched fabric operating in the RC mode maintains a temperature approximately 4.3°C lower than that observed for the pristine fabric. Such axial deformation‐based active 3D dual‐mode thermal management devices not only enable reversible and instantaneous heating/cooling performance but also present practical applicability across diverse platforms, including MEMS devices, buildings, windows, and wearable electronics.

Despite these advantages (Table 1), several practical engineering challenges remain. 3D dual‐mode devices can be susceptible to aerodynamic drag that increases with wind velocity [80]. The resulting wind loads may induce unintended deformation of the 3D architecture and generate localized stress concentrations, which can accelerate interfacial delamination at the substrate–structure interface. As a countermeasure, incorporating porous features—such as hole patterns or micro‐meshes—on the 3D window surface can improve air permeability and reduce the effective wind load [81, 82]. This approach can suppress wind‐induced deformation and alleviate cyclic stress accumulation, thereby improving structural stability and interfacial reliability under outdoor conditions [83]. In addition, outdoor exposure introduces environmental fouling by particulates (e.g., fine dust) and moisture [84]. Such contamination can alter the surface's optical response—e.g., reducing solar absorption and mid‐IR emissivity—thereby degrading thermal management performance and long‐term stability [85]. Surface engineering approaches, including superhydrophobic coatings combined with nano/micro‐texturing, can reduce pollutant adhesion and facilitate removal via rainfall or condensation, provided that the feature scale and morphology are optimized to avoid adverse optical scattering while preserving the radiative cooling functionality [86, 87]. Last, cyclic deformation together with prolonged UV exposure can cause cumulative mechanical fatigue and photo‐degradation in PDMS‐ and PET‐based architectures, potentially leading to permanent set or fracture at highly strained regions [88]. To address this, stimuli‐responsive structural supports (e.g., SMA/SMP‐assisted frames) and high‐recovery elastomers can be considered to improve shape retention and reduce reliance on external actuation. Geometric stress‐relief designs (e.g., rounded cut termini) can further mitigate stress concentrations. Future work should include systematic reliability evaluations—such as cyclic strain retention, residual strain, and quantitative stress mapping supported by finite element analysis (FEA)—to validate long‐term structural integrity [89].

**TABLE 1:** Summary of mechanisms, materials, thermal performance, and applications of radiative 3D dual‐mode thermal management devices utilizing active axial deformation.

Reference No.	Materials		Performances		Applications
	SH	RC	SH	RC	
[67]	Carbon black paint	Ag/PDMS, Ag/SU‐8	5.3°C above ambient temperature on the rooftop	−11.0°C sub‐ambient temperature on the rooftop	MEMS, HVAC
[78]	ITO	Glazing	3.9°C above ambient temperature	−3.3°C sub‐ambient temperature	HVAC
[79]	Cotton yarn with trapped air	TiO_2_/PDMS	Thermal resistance of 0.06 m^2^K/W	−4.3°C sub‐ambient temperature	Textiles, Wearable electronics
[90]	VO_2_ in the insulating phase with Ag/ITO	VO_2_ in the metallic phase with BaF_2_	1.3°C above ambient temperature	−7.9°C sub‐ambient temperature	HVAC
[91]	Transparent hydrogel with Ag NWs	Translucent hydrogel with PDMS cover	0.5°C above ambient temperature	−2.0°C sub‐ambient temperature (Cooling)	HVAC

### 3.2 Flipping

**FIGURE 3:**
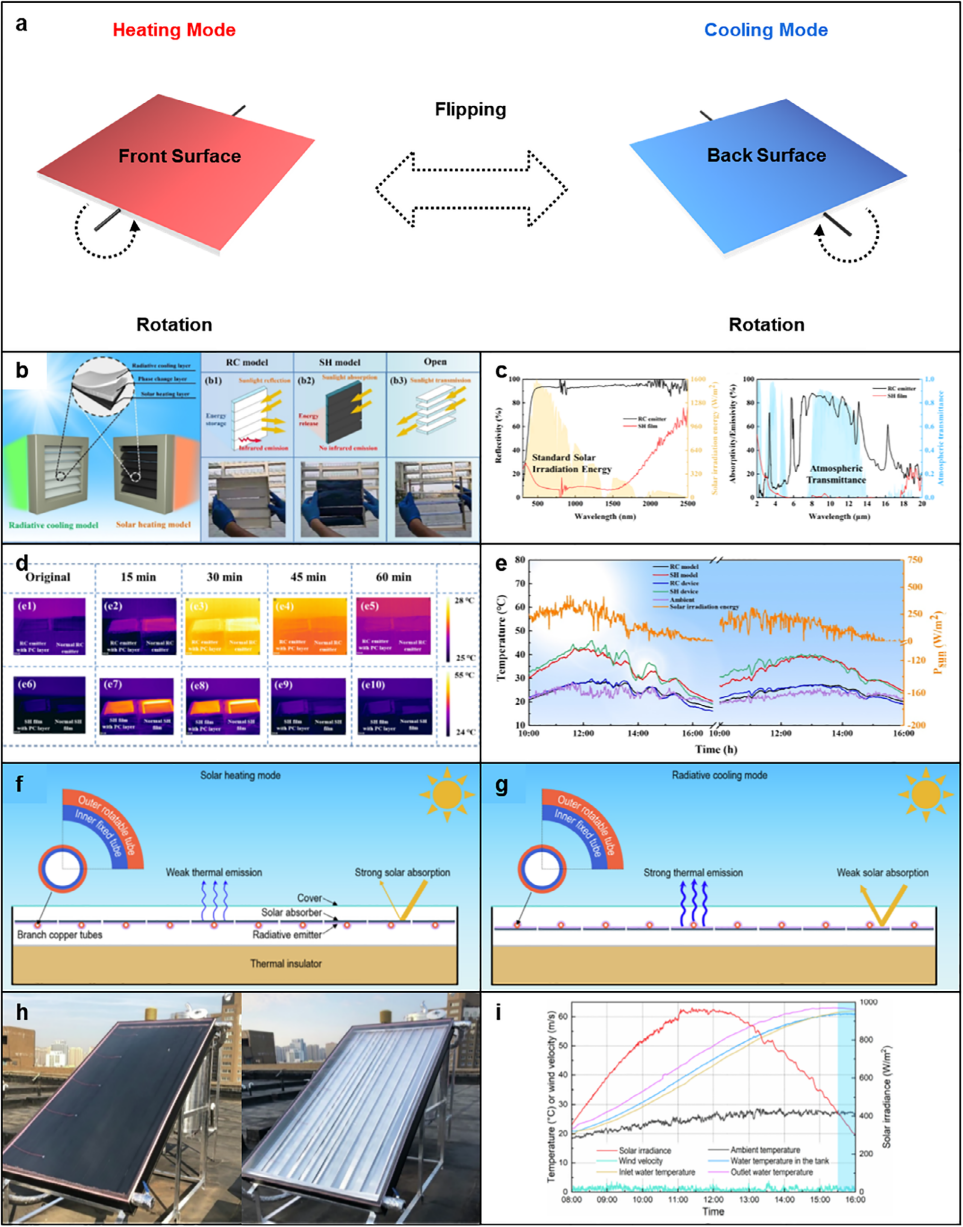
a) Schematic illustration of radiation‐based 3D dual‐mode thermal management devices operated by active flipping. b) Schematic of flipping‐based 3D dual‐mode thermal management devices. c) Optical properties of SH and RC layers. d) IR images comparing PC‐integrated SH and RC devices with individual SH and RC layers. e) Outdoor thermal performance of the 3D devices. Reproduced with permission [92]. Copyright 2023, ACS Publications. Schematic of flipping‐based 3D dual‐mode integrated f) water heating and g) cooling systems. h) Photographs of heating and cooling devices. i) Experimental thermal performance of the integrated systems. Reproduced with permission [93]. Copyright 2022, Elsevier.

Another active strategy for switching is the flipping approach, in which the SH and RC layers are fabricated in a Janus‐like bilayer configuration, and device operation is controlled by flipping the structure to expose either surface (Figure 3a). The device illustrated in Figure 3b adopts a multilayer configuration: the top layer is an SE designed to maximize the solar reflectance [92]. It is fabricated by embedding rare‐earth silicates into a fluorocarbon resin matrix, with an Ag–Al alloy sheet serving as a solar reflector. The middle layer incorporates a phase‐change (PC) film to provide heat storage functionality, whereas the bottom layer performs SH through effective solar absorption. The SE achieves a high solar reflectance and selective emissivity within the atmospheric transparency window, whereas the SH film, comprising an anti‐reflection coating, absorption layer, and a substrate, is optimized to maximize the light‐harvesting performance. The PC layer, fabricated by embedding dodecanol in a polyvinylidene fluoride matrix, further provides thermal energy storage and buffering capability. Figure 3c shows the optical characteristics of the device. The SE exhibits an *ε_LWIR_* of 81% within the atmospheric window (39% outside it) and a solar reflectance of 92%, confirming its RC capability. In contrast, the SH film demonstrates a solar absorptance of 90% and an IR emissivity of only 1%, validating its role as a highly efficient solar absorber. The device operates via a blade‐type flipping mechanism, alternately exposing the SE and SH films to the external environment to switch between the RC and SH modes. Specifically, when the SH film is outward‐facing, it effectively absorbs the solar radiation to induce heating, whereas the exposure of the SE activates RC through its selective emissive properties. This switching strategy, although structurally simple, enables rapid mode transitions and demonstrates the ability to actively adapt to real environmental variations. Comparative temperature measurements between devices incorporating both SH/RC layers with a PC film and those containing only a single SH or RC layer revealed that, under irradiation, the surface temperature of the conventional RC emitter was higher than that of the RC emitter integrated with the PC film. After the illumination was turned off, the internal temperature of the conventional RC emitter decreased rapidly, and the temperature difference between the two nearly disappeared. A similar effect was observed in the SH film, confirming that the PC layer moderates both the heating and cooling rates, thereby facilitating the energy storage function of the 3D active thermal management device (Figure 3d). As shown in Figure 3e, the device achieves a maximum cooling effect of approximately 7°C below the ambient temperature in the RC mode and heating effect of up to 18°C in the SH mode. These results clearly demonstrate that the device functionality extends beyond simple temperature switching, offering a high‐efficiency energy‐saving solution capable of actively adapting to seasonal and environmental variations.

Another flipping mechanism is demonstrated in Figure 3f,g, which depict the main structural components and optical characteristics of the device [93]. The BA exhibits a solar absorptance of 95.1% and an IR emissivity of 8.8%, confirming its excellent SH capability. In contrast, the SE demonstrates a solar absorptance of 13.5% and an emissivity of 92.7%, validating its RC functionality. In addition, the device is equipped with a polyethylene film to suppress convective heat loss from the top surface and copper tubes to efficiently transfer the generated heat. As shown in Figure 3h, this device adopts a flat‐plate configuration combining copper tubes and an insulating layer. It is integrated with a rotatable double‐sided panel that allows switching between SH and RC simply by flipping the panel. During the daytime, solar energy is absorbed by the surface, whereas at night, heat is dissipated via the radiative surface. Because of this simple structure, the required function can be selected quickly and conveniently in response to the environmental conditions. Figure 3i presents the measured SH/RC performance recorded on October 20, 2019, in Hefei, China. During the eight‐hour experiment, the total incident solar energy was 39.4 MJ, with an average ambient temperature of 24.8°C. In the heating mode, the initial water temperature in the tank increased from 21°C (ambient temperature 18.6°C) to a final temperature of 60.8°C (ambient temperature 26.6°C), corresponding to a thermal efficiency of 51.6% during the experiment. In the cooling mode, at 7:25 a.m. on November 18, 2020, under a solar irradiance of 166.3 W·m^−^^2^, the water temperature gradually decreased from 17.4°C to a minimum of 14.3°C. This performance was influenced by the unfavorable local conditions for daytime RC (low altitude of ∼30 m and humid climate). By contrast, in Boulder (altitude > 1600 m, arid climate), the authors (M. Hu et al.) reported an average cooling power of 45 W·m^−^^2^ during midday hours (12:00–14:00) under a solar irradiance of 952 W·m^−^^2^. These results demonstrate that the flipping‐based device can perform both SH and RC and highlight its potential as a high‐efficiency thermal management platform that can directly heat or cool water to flexibly respond to seasonal and temporal demand variations.

While the devices proposed in this study can enhance latent‐heat buffering by increasing PCM loading (Table 2), PCM leakage (up to ≈10% weight loss) remains a major engineering limitation that may compromise shape stability and long‐term performance. In addition, although the dodecanol‐based phase‐change window (15 C–25°C) is suitable under specific conditions, further tuning is required for climates with pronounced diurnal and seasonal variability. Beyond bulk poly(vinylidene fluoride) blending, porous confinement scaffolds (e.g., CNT networks) could improve PCM retention via capillary confinement and adsorption [94, 95]; however, such supports may alter heat‐transfer pathways and optical responses, necessitating co‐optimization of filler fraction, dispersion, and layer thickness. Broadening the operating range via multi‐PCM mixtures with staggered melting points is also promising, provided that material/structural designs suppress phase separation and ensure cycling stability [96, 97]. Moreover, because RC emitters and SA films are susceptible to soiling‐induced optical degradation, surface‐protection strategies (e.g., antifouling coatings) that preserve atmospheric‐window (8–13 µm) emissivity are important for long‐term deployment. Since the current system relies on manual rotation (explicitly described as a prototype), the durability of moving components becomes a key challenge when transitioning to automated actuation for building‐scale integration [98]. The use of a single 120 L tank as both heating and cooling reservoir may also cause incomplete thermal separation during mode transitions, leading to thermal lag and mixing losses that reduce effective energy delivery [99, 100]. These limitations could be mitigated by closed‐loop actuation systems that adjust panel angles based on real‐time solar irradiance and indoor demand (or temperature set points). In parallel, rotating shafts and tube junctions exposed to moisture and dust warrant mechanistic studies of friction‐ and corrosion‐driven degradation, supported by accelerated lifetime testing (cyclic operation, damp‐heat/salt‐spray, and fouling) [101]. Finally, a decoupled dual‐tank design for separate hot/cold storage would reduce intermixing losses and improve response, enhancing overall field performance [102].

**TABLE 2:** Summary of mechanisms, materials, thermal performance, and applications of radiative 3D dual‐mode thermal management devices utilizing active flipping motions.

Reference No.	Materials		Performances		Applications
	SH	RC	SH	RC	
[103]	Commercial SH Film (Sanming, China)	Ag–Al alloy sheet/Fluorocarbon resin with rare‐earth silicate	*A_solar_*: 90%, *ε_LWIR_*: 1%	*R_solar_*: 92%, *ε_LWIR_*: 81%	HVAC
[104]	Black coating on the Al sheet	Commercial RC Film (Ningbo Radi‐Cool Advanced Energy Technologies, China)	A temperature increase of 34.2°C in the water tank	A temperature decrease of 3.1°C in the water tank	HVAC
[105]	MXene film	PDMS/ Ag film	757 W/m^2^	56 W/m^2^	HVAC
[106]	Cs‐doped WO_3_ NPs	Glass	4°C above ambient temperature	−9°C sub‐ambient temperature	HVAC

## 4 Passive‐Type 3D Dual‐Mode Thermal Management Devices

### 4.1 Axial Deformation

**FIGURE 4:**
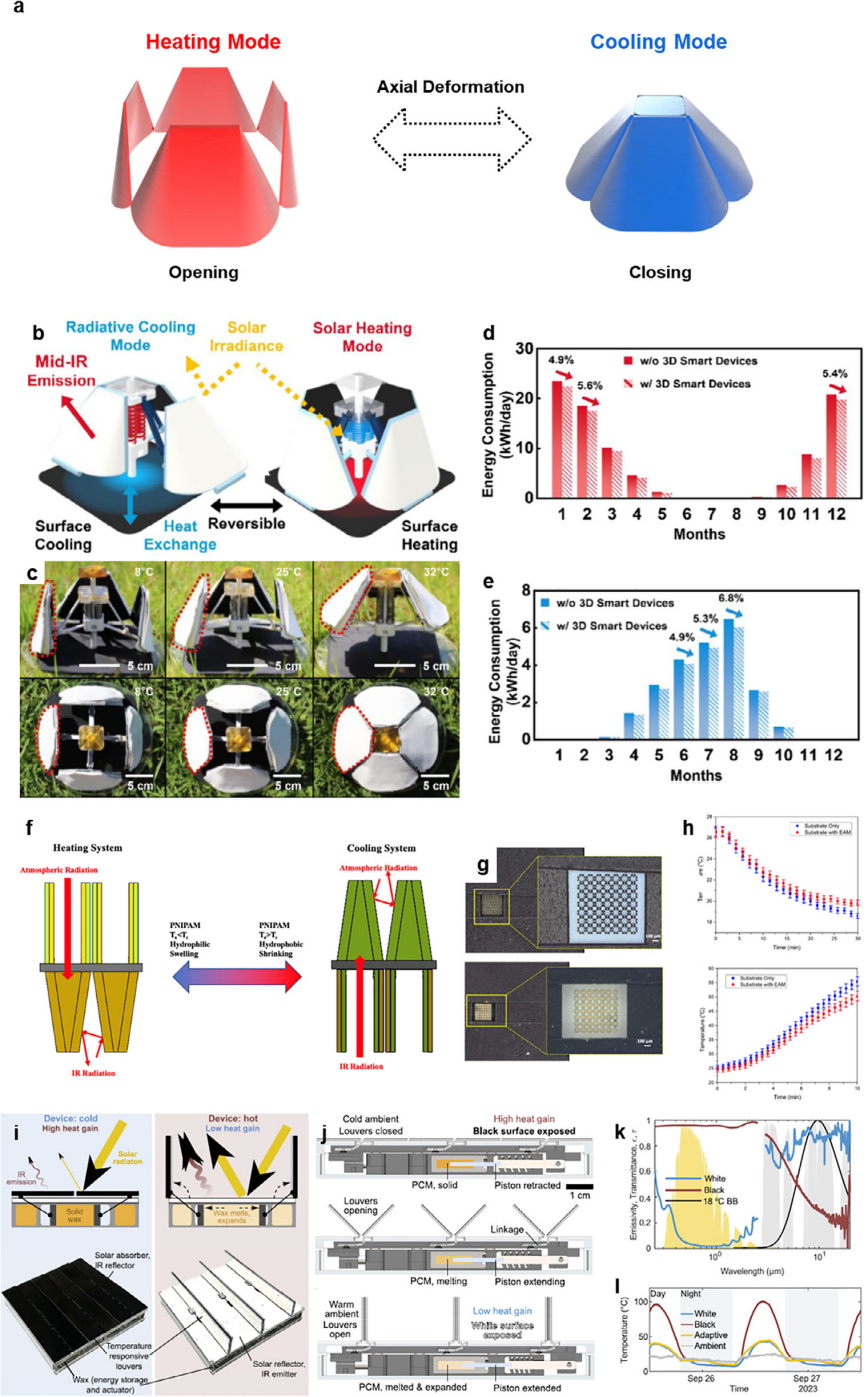
a) Schematic illustration of radiation‐based 3D dual‐mode thermal management devices actuated by passive axial deformation. b) Schematic of passive axial deformation‐based 3D dual‐mode thermal management devices. c) Photographs of devices operating in heating and cooling modes. d) Comparative heating performance with and without 3D devices. e) Comparative cooling performance with and without 3D devices. Reproduced with permission [92]. Copyright 2025, Wiley. f) Schematic illustration of polymer‐based 3D dual‐mode thermal management devices. g) Photographs showing structural transitions between heating and cooling modes. h) Experimental thermal performance of the devices under heating and cooling conditions. Reproduced with permission [93]. Copyright 2021, Wiley. i) Schematic of PCM‐based 3D dual‐mode thermal management devices. j) Operating principles of the reversible switching mechanism triggered by temperature. k) Optical properties of the solar absorber and SE. l) Comparison of thermal properties between dual‐mode devices and single‐mode films [107]. Reproduced with permission. Copyright 2024, Elsevier.

In contrast to active‐type devices, passive 3D dual‐mode thermal management devices represent a promising strategy for maximizing the energy‐saving efficiency, as they can spontaneously and reversibly switch between SH and RC without external energy consumption [92]. In particular, these devices possess the advantage of adaptive temperature regulation in response to environmental variations, thereby continuously maintaining a stable performance without the need for additional control (Figure 4a). Among such passive thermal management devices, Figure 4b illustrates a system that employs shape memory alloy (SMA)‐induced axial deformation to achieve reversible switching between SH and RC. The BA for the heating mode is fabricated with a carbon‐black‐based coating, which provides high solar absorptance and enables effective heat storage. The SE for the cooling mode is fabricated using an Al_2_O_3_/PDMS composite film, which exhibits high solar reflectance while delivering strong emissivity within the atmospheric transparency window (8–13 µm). This configuration minimizes the solar heat gain during the daytime while maximizing the radiative heat dissipation into outer space. Figure 4c illustrates the structural switching mechanism based on the temperature‐responsive behavior of the SMA. As the ambient temperature rises, the SMA elongates from approximately 3 to 5 cm within the range of 10 C–40°C, causing the wing‐like structure to close. In this state, the SE layer is exposed outward, whereas the BA is shielded, thereby activating the RC mode. Conversely, as the temperature decreases, the SMA contracts from approximately 3 to 2.5 cm over the range of 30 C–0°C, reopening the wings and directly exposing the BA to sunlight, thus enabling efficient SH. During this reversible process, the wing angle changes between approximately 45° and 90°. Such structural transitions are spontaneously triggered by temperature variations, without the need for external power supply or control units. In this study, the authors analyzed the theoretical energy‐saving potential of the proposed 3D device using typical meteorological data for a standard building located in Daejeon, Republic of Korea (36.4°N, 127.4°E). The results revealed that the device could reduce heating energy consumption in winter (December–February) by approximately 4.9%–5.6% (Figure 4d) and cooling energy consumption in summer (June–August) by approximately 4.9%–6.8% (Figure 4e). Furthermore, annual analyses conducted for other regions also demonstrate similarly favorable energy‐saving outcomes. In experimental investigations of the SH mode, the device demonstrated a maximum temperature rise of approximately +51.3°C above the ambient temperature and a solar‐to‐thermal conversion efficiency exceeding 65%. In the RC mode, a temperature reduction of −6.1°C and cooling power of approximately 36 W/m^2^ were achieved. These results clearly indicate that the device does not function merely as a simple heating–cooling switch, but as a high‐efficiency energy‐saving platform capable of adaptively responding to diverse environmental conditions, including seasonal variations and diurnal fluctuations.

Figure 4f shows a passive 3D dual‐mode thermal management device fabricated using a thermoresponsive hydrogel based on poly(N‐isopropylacrylamide) (PNIPAM) [93]. PNIPAM has a low critical solution temperature (LCST) of approximately 32°C; below this temperature, it absorbs water and remains in a swollen state, whereas above the LCST, it undergoes dehydration and shrinkage, transitioning to a hydrophobic state. Based on this behavior, open‐flower and closed‐flower configurations were designed, corresponding to radiative heating and RC functions, respectively. The experimental results showed that the emissivity difference between the two structural states reached approximately 35%. In particular, the closed configuration exhibited a high emissivity of up to ∼95% within the atmospheric transparency window (8–13 µm), demonstrating that this structure can effectively realize both SH and RC performance. Figure 4g illustrates the reversible structural switching mechanism of the device. This device is fabricated in a micro‐pyramid/petal configuration using the 4D printing‐based two‐photon polymerization technology. Each unit structure comprises inner and outer layers with low and high crosslinking densities, respectively. When the temperature rises above 32°C, the inner layer contracts to a greater extent than the outer layer, causing the entire structure to bend inward and form a closed‐flower configuration. Conversely, when the temperature decreases, the inner layer reswells, allowing the structure to revert to its original open‐flower state. This structural transition occurs rapidly, within approximately one minute, and operates autonomously in response to environmental temperature changes without the need for external power or control units. Figure 4h presents the temperature response characteristics associated with the open–closed structural transition. The radiative properties vary distinctly with the structural change, and the experiments confirmed a maximum heating/cooling output of approximately 185 W·m^−^^2^. Specifically, in the SH mode, a temperature rise of approximately +5°C above the ambient temperature was observed, whereas in the RC mode, a reduction of approximately −6 C to −7°C was achieved. This performance is markedly superior to that of single‐function devices, indicating the capability for bidirectional energy control using a single structure. Furthermore, when the device was scaled up to the centimeter level (2.54 × 2.54 cm^2^), the results showed reduced temperature fluctuations and improved response speed when compared with that of the control group, thereby demonstrating its potential as a passive energy‐saving solution for systems such as buildings and vehicles.

The device proposed in Figure 4i implements dual‐mode functionality based on the deformation of a 3D louver structure [107]. This system achieves reversible switching between the SH and RC modes by exploiting the volume change associated with the melting and solidification of hexadecane, a phase‐change material (PCM). In the SH mode, the PCM remains below its melting point (18.2°C) in the solid state with a lower volume. In this condition, the piston link does not move, leaving the upper BA exposed to enable SH. Conversely, in the RC mode, the PCM liquefies and expands, pushing the piston link upward. This motion opens the louver, exposing the inner white high‐emissivity layer and the rear aluminized Mylar reflective layer to the outer environment, thereby activating RC. This 3D structural transition occurs rapidly with a small temperature difference of within ∼3°C and exhibits low hysteresis, allowing the device to respond sensitively to typical diurnal temperature fluctuations (Figure 4j). The black chrome‐coated aluminum used as the solar absorber demonstrates a high absorptance of approximately 96% in the solar spectrum (0.2–2.5 µm) and an emissivity of approximately 30% in the atmospheric transparency window (8–13 µm), confirming its effective SH performance. In contrast, the SE based on BaSO_4_ exhibits a low absorptance of ≈5% in the solar spectrum and high emissivity of ≈88% in the atmospheric window, validating its capability for RC (Figure 4k). Outdoor testing results presented in Figure 4l show that the temperature performance of the proposed device is closer to the ideal indoor temperature than that of the non‐switching control samples under both daytime and nighttime conditions. At night, the PCM remains in the solid state, keeping the louvers closed and exposing the BA layer. This configuration suppresses IR emissions and prevents excessive cooling. As a result, the adaptive device and black surface exhibit temperature reductions of approximately 8.6°C and 9.1°C below the reference temperature, respectively, whereas the white surface shows the largest cooling effect, achieving a decrease of up to 11.3°C. Conversely, during the daytime, the PCM liquefies, opening the louvers and exposing the high‐emissivity white surface, thereby suppressing overheating. Under these conditions, the black surface reaches a maximum temperature of +82.7°C, indicating severe overheating, whereas the adaptive device and white surface remain at +27.5°C and +25.7°C, respectively, confirming their effectiveness in reducing the cooling load.

Despite these advantages (Table 3), transitioning from single‐device performance validation to practical building‐exterior applications necessitates a quantitative analysis of complex interactions within large‐scale arrays. Specifically, mutual shading and inter‐unit radiative exchange can change the effective solar absorption/reflection as well as the array‐level radiative exchange to the sky (via view‐factor reduction and re‐absorption between neighboring units), thereby influencing system‐level energy yield [108]. In addition, the increased aerodynamic resistance inherent in 3D architectures may compromise structural integrity under high‐wind conditions. Environmental exposure further introduces soiling and moisture condensation on moving/functional components, which can degrade thermo‐optical properties (e.g., solar selectivity and mid‐IR radiative performance) and reduce actuation reproducibility over time, highlighting the need for rigorous long‐term reliability validation [30]. To mitigate these constraints, optical–geometric modeling that incorporates regional solar altitude and azimuth trajectories should be performed to optimize module pitch and layout and minimize inter‐module interference [109, 110]. At the array level, design refinements that control wing opening angles and morphologies are also required to preserve a sufficient sky view factor and suppress radiative trapping within the assembly [111, 112]. From a structural standpoint, wind loads can be reduced by aerodynamically dispersive features such as streamlined wing tips or micro‐perforations, while high‐strength composite joints and integrated frame structures can distribute loads across the array and improve overall mechanical robustness under outdoor wind extremes [113, 114]. Although passive switching devices show strong potential for autonomous adaptation to environmental changes without external power, many reported systems remain constrained by operating windows that are tightly coupled to the intrinsic transition temperatures of their constituent materials. For example, PNIPAM‐based hydrogel devices typically switch near ≈32°C, while hexadecane‐based PCM systems trigger around ≈18°C, which limits tunability across diverse climates and user‐defined comfort targets. Moreover, narrow temperature windows can cause frequent, undesired switching under rapid fluctuations, whereas pronounced hysteresis can delay transitions and compromise timely thermal regulation. Addressing these limitations requires a materials toolbox that enables precise tuning of activation thresholds at the molecular/compositional level. In SMAs, the transformation temperature range can be tailored by adjusting the Ni–Ti atomic ratio and heat‐treatment conditions; in PNIPAM systems, the LCST can be shifted toward target temperatures through copolymerization of hydrophilic/hydrophobic monomers and rational network design [115, 116, 117]. Beyond material‐level tuning, hybrid actuation strategies can be introduced at the structural level, where geometrically applied pre‐stress or pre‐strain shifts the effective switching conditions and stabilizes operation against environmental perturbations [118, 119]. Finally, integrating multiple PCMs or responsive materials with staggered transition temperatures into an array configuration can enable multistage thermal regulation with incremental responses across a broader temperature spectrum, provided that the material/structural design suppresses phase segregation and maintains cyclic stability over long‐term operation.

**TABLE 3:** Summary of mechanisms, materials, thermal performance, and applications of radiative 3D dual‐mode thermal management devices utilizing passive axial deformation.

Reference No.	Materials		Performances		Applications
	SH	RC	SH	RC	
[120]	Carbon black paint	Al_2_O_3_/PDMS	45.3°C above ambient temperature	−6.1°C sub‐ambient temperature	HVAC
[93]	PNIPAM in the closed‐flower state	PNIPAM in the open‐flower state	5°C above ambient temperature	−6–7°C sub‐ambient temperature	MEMS, HVAC
[107]	Black chrome‐coated Al sheet	BaSO_4_ paint	*A_solar_*: 96%, *ε_LWIR_*: 30%	*A_solar_*: 5%, *ε_LWIR_*: 88%	HVAC
[121]	Blue Ti film or Black Cr film	Silica particles with a PP substrate	20.7°C above ambient temperature	−9.9°C sub‐ambient temperature	HVAC

### 4.2 Bending

**FIGURE 5:**
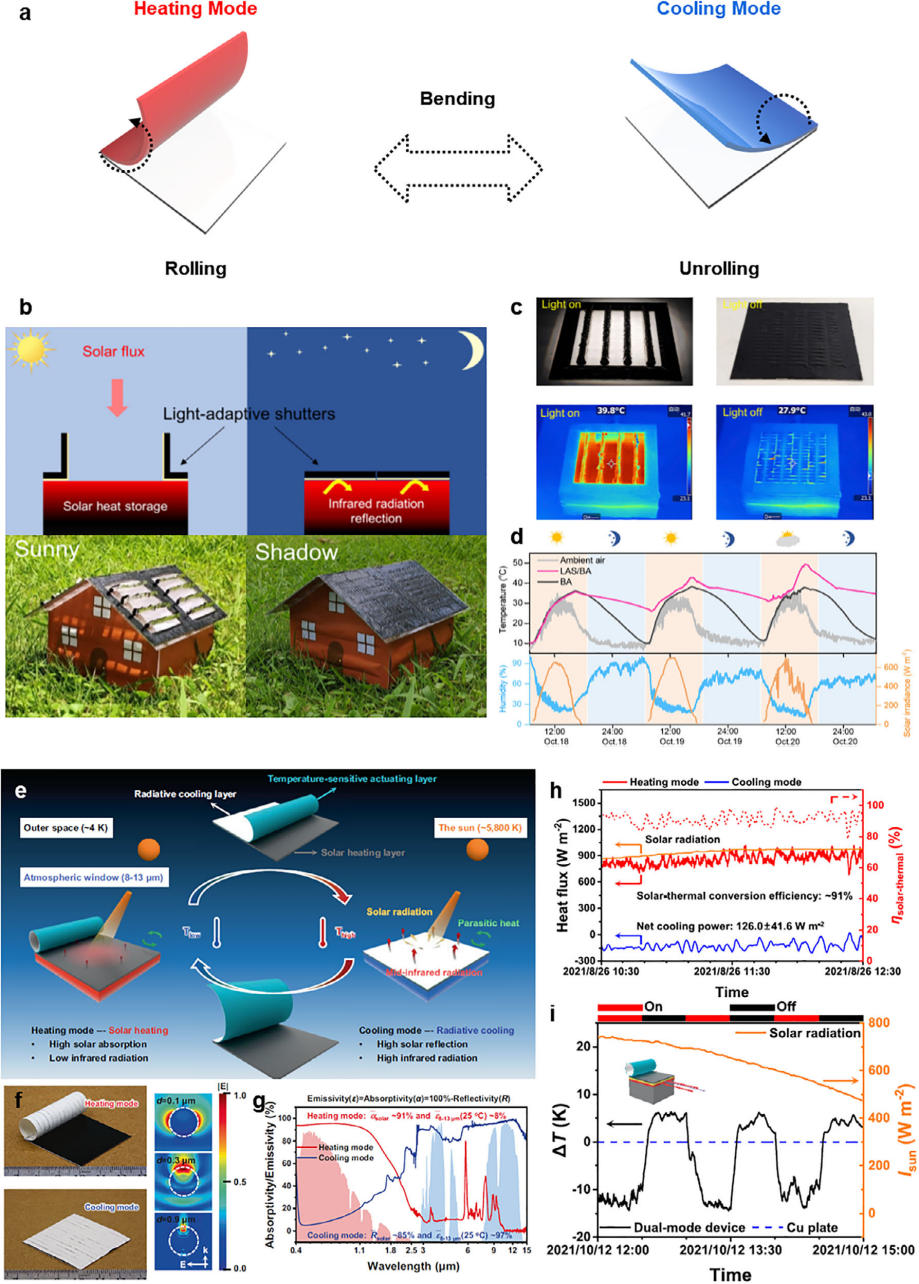
a) Schematic illustration of radiation‐based 3D dual‐mode thermal management devices actuated by passive bending. b) Schematic of passive bending‐based 3D dual‐mode thermal management devices. c) Photographs and IR images of the devices in the heating and cooling modes. d) Comparative thermal performances with and without 3D devices. Reproduced with permission [122]. Copyright 2021, Elsevier. e) Schematic of bending‐based 3D dual‐mode thermal management mechanism. f) Photographs of the devices in the heating and cooling modes, along with cross‐sectional views of the light field around TiO_2_ spheres of different diameters. g) Optical properties of the devices in the heating and cooling modes. h) Measured heat flux of the devices. i) Comparison of thermal performance of the device with the Cu plate. Reproduced under terms of the CC‐BY license [92]. Copyright 2022, Q. Zhang et al., published by Springer Nature.

Another passive deformation approach is the bending mechanism (Figure 5a). For example, Figure 5b illustrates a case in which a film that undergoes bending deformation in response to light stimulation is employed to realize a passive‐type 3D dual‐mode thermal management device. This device contains three thin‐film layers. The CNF/CNT layer exhibits a solar absorptance exceeding 95%. The ethyl cellulose (EC)/CNT layer functions as the actuator, inducing structural bending in response to temperature changes [122].

The Ag layer serves as a reflective layer with an IR reflectance of ≈97%, suppressing radiative losses and enhancing the cooling performance. Thus, the device integrates a BA (graphite sheet/polypyrrole) absorber with an absorptance of solar irradiation (*A_solar_*; 95%–98%) and a selective absorber (titanium‐coated film) to achieve heating via solar absorption and cooling through radiative loss. The film deformation arises from a thermal expansion mismatch caused by the photothermal effect. The CNF/CNT layer exhibits a negative thermal expansion coefficient (−378 × 10^−^^6^ K^−^^1^), whereas the EC/CNT layer shows a positive coefficient (+13 × 10^−^^6^ K^−^^1^), resulting in a large mismatch between the two layers. Upon solar irradiation, the photothermal effect of the CNTs induces a temperature rise, which amplifies the thermal expansion mismatch and rapidly triggers bending deformation of the film. This deformation occurs within approximately 0.7–3.5 s, with bending angles of 90–360°. In addition, higher humidity accelerates the volume changes caused by the hydrophilic nature of the CNF, which promotes active adsorption and desorption of the water molecules, thereby improving the response speed. Based on this principle, no deformation occurs at lower temperatures, exposing the black CNF/CNT absorbing layer and enabling the SH mode. Conversely, when the temperature rises, the film actively bends, exposing the Ag reflective layer, which facilitates heat dissipation and switches the device into the RC mode. An analysis of the solar heat‐storage performance revealed that the passive‐type 3D dual‐mode thermal management device exhibited excellent functionality. When a BA (polypyrrole‐modified graphite sheet) was employed as the absorbing layer, the temperature increased to approximately 50°C under 3 h of solar irradiation (1,000 W/m^2^). However, under nighttime conditions (−20°C ice–salt bath), the system underwent rapid cooling to −8.5°C owing to strong radiative losses. In contrast, when an SA (titanium‐coated film) was used, the temperature reached up to 57°C and maintained a relatively stable value of ≈17°C at night, which was attributed to reduced radiative losses. Notably, when integrated with the 3D device, both absorbers demonstrated significantly enhanced heat storage efficiency. The BA‐ and SA‐integrated systems stabilized at ≈19°C and ≈21°C, respectively, which were 27°C and 4°C higher than the corresponding temperatures of their single‐layer counterparts. These results demonstrate that the device not only provides shading effects but also structurally suppresses nocturnal radiative losses, thereby effectively retaining the stored thermal energy. Theoretical calculations further supported these findings, showing that in a −20°C environment, the radiative loss powers of the BA and SA were 224 W/m^2^ and 21 W/m^2^, respectively, and their integration with the device reduced these values significantly to 10 W/m^2^ and 7 W/m^2^ (Figure 5c). An outdoor validation confirmed the same trend: a house model equipped with the device responded adaptively to the environmental changes, opening automatically under sunlight and fully closing in shaded or nighttime conditions. During a three‐day continuous outdoor test, the BA‐only system exhibited diurnal temperature fluctuations nearly synchronized with those of the ambient environment, resulting in net heat storage close to zero or even a negative value. In contrast, the system integrated with the device showed a gradual increase in the minimum nighttime temperature from 10°C to 35°C, which was approximately 23°C higher under the same conditions. This clearly demonstrates that the device achieves stable and highly efficient heat storage performance even in real outdoor environments (Figure 5d).

Another example is shown in Figure 5e, where the device demonstrates efficient SH performance in the heating mode. The exposed nano‐Cr black aluminum plate absorbs ≈91% of the solar radiation and emits 8% of the IR emission. In contrast, the poly (4‐methyl‐1‐pentene) (PMP) film embedded with TiO_2_ nanoparticles reflects approximately 90% of the solar radiation and exhibits a high emissivity of 97% in the atmospheric transparency window, thereby enabling RC functionality [92]. In addition, a two‐way shape memory polymer (2 W SMP) serves as an actuator that induces reversible bending deformations, enabling switching between the SH and RC modes. As shown in Figure 5f, the transition between the SH and RC modes in the 3D device is achieved because of a length mismatch between the 2 W SMP and RC layer. In the heating mode (≈20°C), the 2 W SMP elongates whereas the RC layer exhibits a negligible length change, leading to the accumulation of interfacial stress. Consequently, the structure bends into a coiled configuration, exposing the underlying solar absorber layer. In this stage, efficient SH is realized through blackbody absorption. In contrast, in the cooling mode (≈40°C), the SMP contracts, releasing the interfacial stress and allowing the SMP/RC layer to flatten, thereby covering the heating layer. In this stage, the RC layer reflects more than 90% of the incident solar radiation. This high reflectance arises from multiple scattering and internal reflection caused by the refractive index mismatch between TiO_2_ (n > 2.39) and the PMP matrix (n ≈1.46), as well as the multiple Mie resonance effects induced by TiO_2_ nanoparticles of varying sizes, which together enable broadband solar reflection across the 0.3–2.5 µm spectrum. Thus, the RC layer simultaneously achieves a solar reflectance greater than 90% and an MIR (8–13 µm) emissivity of approximately 96%, thereby enabling effective RC performance (Figure 5g). As shown in Figure 5h, in the heating mode, the output steadily increases with rising solar irradiance, reaching ≈958.7 W/m^2^, whereas the solar‐to‐thermal energy conversion efficiency remains stable at ≈91%. In contrast, in the cooling mode, when the solar irradiance exceeds 850 W/m^2^ near noon, an average RC power of ≈126.0 W/m^2^ is observed. Furthermore, outdoor tests confirmed that in comparison with a bare copper plate, the device achieves a temperature rise of ≈6 K in the heating mode and temperature reduction of ≈15 K in the cooling mode (Figure 5i). These findings demonstrate that the device delivers effective heating in winter and pronounced cooling in summer, thereby delivering year‐round energy‐saving effects.

These approaches show zero energy consumption during transitions (Table 4), but SMP‐based 3D thermal management architectures can exhibit reduced stiffness near the transition temperature, where the elastic modulus drops sharply [123]. This thermal softening makes the structures more vulnerable to wind‐induced mechanical instabilities—such as creep, buckling, and fluttering—under outdoor wind loads [124, 125]. Such distortions compromise the geometric fidelity of the target configuration, thereby impairing the repeatability and precision of mode switching and reducing structural robustness under cyclic loading [126]. To mitigate these issues, viable strategies include implementing mechanical interlocking features to stabilize the structure at the target temperature and/or integrating hybrid support–fixation mechanisms assisted by SMA auxiliary actuators. In particular, an auxiliary load‐bearing skeleton can redistribute the load path while the SMP is softened, improving shape retention and maintaining stable thermal management operation under strong‐wind conditions [119, 127]. For outdoor deployment, long‐term durability requires systematic datasets and protection strategies against multiple environmental stressors, including UV radiation, moisture, and particulate fouling. Polymer‐based SMPs and optical films are susceptible to UV‐driven photodegradation, which can deteriorate mechanical properties over time. In addition, moisture ingress may weaken interfacial adhesion and promote delamination, while humidity‐dependent plasticization can introduce variability in deformation kinetics and compromise cyclic reproducibility [124, 128]. As a robust approach, transparent inorganic barrier layers (e.g., Al_2_O_3_ or SiO_2_) deposited via atomic layer deposition can be incorporated to suppress moisture/oxygen permeation [129, 130]. To further mitigate fouling‐induced optical degradation, these barriers can be functionalized with anti‐soiling surface treatments—such as low‐surface‐energy coatings (e.g., siloxane or fluorinated layers) or carefully designed micro/nanoscale texturing—provided that the surface architecture is optimized to minimize optical scattering and maintain transparency [131, 132].

**TABLE 4:** Summary of mechanisms, materials, thermal performance, and applications of radiative 3D dual‐mode thermal management devices utilizing passive bending motions.

Reference No.	Materials		Performances		Applications
	SH	RC	SH	RC	
[122]	Graphite sheet/Polypyrrole	Ti‐coated film/Ag	224 W/m^2^	21 W/m^2^	HVAC
[92]	Nano‐Cr black Al sheet	TiO_2_/PMP	958.7 W/m^2^	126.0 W/m^2^	HVAC
[133]	Zn sheet	hBN nanoplates with the polymer styrene ethylene butylene styrene	12.7°C above ambient temperature	−8.3°C sub‐ambient temperature	HVAC
[134]	CNT or Cs‐doped WO_3_ NPs	Polyethersulfone/ N‐methyl‐2‐pyrrolidone/ Al_2_O_3_	16.8°C above ambient temperature	−8.8°C sub‐ambient temperature	HVAC

## 5 Summary and Outlook

While high‐performance static‐mode devices, including single‐mode SH and RC systems, have demonstrated substantial energy‐saving potential, they remain fundamentally constrained by their limited adaptability to dynamically changing thermal environments, particularly those characterized by large diurnal temperature fluctuations or simultaneous heating and cooling demands. In this context, 3D dual‐mode thermal management devices (i.e., adaptive‐mode devices) have emerged as a compelling alternative. Their defining design principle is the reversible reconfiguration of functional SH and RC layers, enabling dynamic modulation of solar absorptance/reflectance (0.3–2.5 µm) and MIR emissivity (8–13 µm) within a single platform. Adaptive‐mode devices achieve rapid mode switching through externally triggered structural transformations. Representative examples include axial‐deformation‐based devices composed of silicone elastomers and stretchable fibers, which allow tunable control over *R_solar_* and *ε_LWIR_* and have been demonstrated in electronics, textiles, and smart‐window applications. Likewise, flipping‐based configurations, which rely on louver rotation, have illustrated the feasibility of bidirectional thermal regulation in architectural envelopes. By contrast, passive systems operate autonomously without external energy input, thereby representing an intrinsically energy‐efficient strategy. Axial deformation‐based passive devices typically exploit the volumetric response of SMAs or SMPs, whereas bending‐based architectures harness interlayer mismatch in SMPs to induce temperature‐dependent optical and structural reconfiguration.

As summarized in Tables 1, 2, 3, 4, these mechanisms collectively reflect the rapid evolution of 3D dual‐mode thermal management technologies. More importantly, adaptive dual‐mode systems have demonstrated up to a nine‐fold improvement in annual energy‐saving efficiency and net cost savings relative to static single‐mode devices (Table 5), underscoring their potential to redefine the performance ceiling of next‐generation thermal management platforms. Nevertheless, several limitations remain, as follows:

- Repeated structural reconfiguration introduces a critical reliability challenge. Future research must move beyond simple transition‐cycle testing and establish comprehensive reliability protocols that account for coupled environmental stressors, including UV irradiation, humidity, thermal cycling, and long‐term outdoor exposure. This issue is particularly acute for polymer‐based and sublimation‐driven systems, which are vulnerable to photodegradation, creep, and interfacial fatigue. A key research priority is therefore the development of quantitative degradation maps and predictive lifetime models that correlate environmental loading conditions with structural and functional failure.
- Surface fouling remains a major yet insufficiently addressed obstacle to real‐world performance retention. In outdoor settings, the accumulation of dust, moisture, and other contaminants can significantly perturb solar reflectance and MIR emissivity, thereby undermining thermal regulation efficiency. Although antifouling approaches based on polydopamine, TiO_2_, micro/nanostructures, and superhydrophobic coatings offer promising mitigation pathways, their integration into dual‐mode devices raises important trade‐offs with intrinsic radiative performance. Future efforts should therefore focus on establishing multifunctional surface design principles that simultaneously optimize fouling resistance, optical selectivity, and long‐term environmental stability.
- The inherent geometric complexity of 3D architectures renders them mechanically vulnerable under outdoor wind loading. Continuous exposure to aerodynamic drag may induce unintended deformation, mechanical instability, or catastrophic failure, especially in compliant or thermally softened structures. Accordingly, wind‐resilient aero‐mechanical engineering should be recognized as a central research direction. Porous surface designs incorporating hole patterns or micro‐mesh structures could reduce effective wind pressure by improving air permeability, while enhanced interfacial adhesion and mechanical locking schemes may suppress delamination and improve structural robustness. Beyond these passive stabilization approaches, the field would also benefit from the development of hybrid actuation architectures that integrate SMAs, SMPs, and PCMs. Such systems could simultaneously expand the operational temperature window and improve transformation stability, thereby enabling more climate‐adaptive and application‐specific device functionality.
- Large‐area implementation remains an unresolved systems‐level challenge. Although many reported devices exhibit promising performance at the individual unit level, their collective behavior in arrays may be substantially altered by mutual shading, radiative coupling, thermal cross‐talk, and aerodynamic interactions. Addressing this issue requires high‐fidelity multiphysics simulations capable of identifying optimal device spacing, packing density, and geometric configuration at the module and system scales. At the same time, scalable manufacturing strategies, such as roll‐to‐roll processing and modular assembly, must be evaluated in parallel with cost models, maintenance requirements, and life‐cycle assessment frameworks. Only through this integrated systems perspective can the gap between proof‐of‐concept demonstrations and commercially relevant technologies be meaningfully narrowed.

**TABLE 5:** Quantitative techno‐economic and thermodynamic performance comparison between static and adaptive devices.

Reference No.	Mode (Static/Adaptive)	Number of locations (units)	Energy saving (MJ/m^2^·year)	Net cost saving ($/m^2^·year)
[135]	Static	9	17.99	0.792
[136]	Static	15	22.4	0.986
[137]	Static	3	46	2.024
[90]	Adaptive	22	89.5	3.938
[138]	Adaptive	19	99.5	4.378
[139]	Adaptive	11	166.5	7.326

In conclusion, 3D dual‐mode thermal management devices represent a reversibly switchable platform for overcoming the intrinsic limitations of conventional single‐mode SH and RC systems. Yet their future impact will depend not only on maximizing switching performance but also on resolving deeper challenges associated with environmental durability, structural reliability, surface stability, manufacturability, and economic scalability. The next phase of research should therefore shift from isolated material‐ or device‐level optimization toward a holistic design paradigm that integrates optical engineering, mechanical resilience, environmental reliability, and techno‐economic viability. If these challenges can be systematically addressed, radiation‐based adaptive thermal management systems are poised to become a foundational technology for sustainable energy savings across buildings, electronics, and beyond.

## Nomenclature

### Acronyms

3D: Three‐dimensional
AM: Air‐mass
BA: Broadband absorber
CNF: Carbon nanofiber
CNT: Carbon nanotube
EC: Ethyl cellulose
FEA: Finite element analysis
HVAC: Heating, ventilation, and air‐conditioning
ITO: Indium tin oxide
LCST: Low critical solution temperature
LWIR: Long‐wave infrared
MIR: Mid‐infrared
NP: Nanoparticle
PC: Phase‐change
PCM: Phase‐change material
PDMS: Polydimethylsiloxane
PET: Polyethylene terephthalate
PMP: Poly(4‐methyl‐1‐pentene)
PNIPAM: Poly(N‐isopropylacrylamide)
PP: Polypropylene
RC: Radiative cooling
SA: Solar absorbing
SE: Selective emitter
SH: Solar heating
SMA: Shape memory alloy
SMP: Shape memory polymer
UV: Ultraviolet

### Symbols and Expressions

A_solar_
: Averaged solar absorptance
I_b_
: Intensity of blackbody radiation
I_solar_
: Solar irradiance
P_atm_
: Absorbed atmospheric emission
P_cool_
: RC cooling power
P_rad_
: Spontaneously emitted thermal radiation
P_sol_
: Absorbed solar irradiance
R_solar_
: Averaged solar reflectance
T_amb_
: Ambient temperature
ε_LWIR_
: LWIR emissivity
τ_atm_
: Atmospheric transmittance

## Conflicts of Interest

The authors declare no conflicts of interest.

## Data Availability

The authors have nothing to report.
